# Contextual and individual inequalities in breast cancer screening participation and outcomes in Turin (North-West Italy)

**DOI:** 10.1038/s41523-024-00660-4

**Published:** 2024-06-27

**Authors:** Chiara Di Girolamo, Giulio Cammarata, Livia Giordano, Nicolás Zengarini, Elisa Ferracin, Viviana Vergini, Gianluigi Ferrante, Fulvio Ricceri

**Affiliations:** 1 Centre for Biostatistics, Epidemiology, and Public Health (C-BEPH) – Department of Clinical and Biological Sciences, University of Turin, Regione Gonzole 10, 10043 Orbassano Turin, Italy; 2 Cluster Reply - Via Cardinale Massaia 83, 10147 Turin, Italy; 3 SSD Epidemiologia Screening - CRPT, AOU Città della Salute e della Scienza di Torino, Corso Bramante 88, 10126 Turin, Italy; 4 Epidemiology Department, Local Health Unit TO3, Via Sabaudia, 164, 10095 Grugliasco, Italy

**Keywords:** Population screening, Breast cancer, Cancer epidemiology, Epidemiology

## Abstract

Breast cancer incidence and screening participation exhibit an unequal distribution in the population. This study aims to investigate the impact of socioeconomic position (SEP) on three breast screening indicators (participation, recall, and cancer detection rates) among women aged 50–69 in the city of Turin between 2010 and 2019. The study also aims to determine whether contextual factors (deprivation index) or individual factors (educational level) have a greater influence. The data used in this study are sourced from the Turin Breast Screening Program (TBSP) and the Turin Longitudinal Study (TLS). To test the hypothesis and account for the hierarchical structure of the data, multilevel models were used. Both contextual and individual SEP were found to be associated with screening participation. Participation increased with higher levels of deprivation (odds ratio for most deprived: 1.13; 95% CI 1.11–1.16) and decreased with higher educational levels (OR for low educated: 1.37; 95% CI 1.34–1.40). Contextual SEP did not show any association with recall or cancer detection rates, but individual SEP had an impact. Women with lower educational levels had a statistically significant 19% lower odds of being recalled and a statistically significant 20% lower odds of being diagnosed with cancer. Additionally, immigrant women were less likely to participate in screening, be recalled, or receive a cancer diagnosis. Educational level consistently influenced the analyzed screening indicators, while contextual deprivation appeared to have less importance. It is likely that women living in less deprived areas and with higher education have greater access to opportunistic screening.

## Introduction

In Europe, breast cancer accounts for 28.7% of all new cancers and is the first cause of cancer death among women^1^. In Italy, 183,201 new cases and 77,694 deaths from breast cancer were estimated in 2020 and, although breast cancer mortality has declined over the years, it remains the first cause of cancer death among women^2^. According to the data of the screening program *Prevenzione Serena*, in 2021, the detection rate for breast cancer among women 50–69 years in Turin was 7.2‰^3^.

Despite well-known downsizes, such as over-diagnosis, there is an agreed consensus on the effectiveness of secondary prevention through mammography-based screening programs in improving breast cancer outcomes^4^.

The uptake of oncological screenings among women is not evenly distributed across the population, and substantial socioeconomic inequalities have been reported^5,6^. For breast cancer screening, there is evidence that women in lower socioeconomic positions have generally lower incidence but worse outcomes^7,8^; additionally, a lower breast cancer screening uptake among those living in the more deprived areas has been reported in Europe^5,9–11^.

The objective of this study is to investigate, among women aged 50–69 and residents in the city of Turin (Northwest of Italy), the effect of socioeconomic position on three breast screening indicators, namely participation, recall, and cancer detection rates, and to assess whether the contextual or the individual socioeconomic position matters more, after controlling for women’s age, citizenship, and past screening behaviors.

## Results

### Participation rate

For this outcome, 527,987 observations were considered. Table 1 shows the descriptive statistics and the estimates of the univariable logistic models. Overall, adherence to screening was about 60%. The odds of participating in screening was the lowest among women aged 55–59 years (OR 0.89, 95%CI 0.88–0.91) and those from High Migratory Pressure Countries (HMPC) (OR 0.47, 95%CI 0.46–0.48). It was the highest among women who participated regularly in the screening program (OR 3.21, 95%CI 3.14–3.27), those with a low educational level (OR 1.37, 95%CI 1.34–1.40), and among those living in high deprivation areas (OR 1.21, 95%CI 1.18–1.23). The Wald test confirms the hypothesis that deprivation presents a random effect (*p* value <0.001), which means that variability in the participation rate exists among census blocks with a similar deprivation level. The estimates of the random slope multilevel model reveal that both individual and contextual characteristics impact screening participation (Table 2). After controlling for all variables, the level of deprivation of the area the woman lives in showed a direct gradient, though smaller than the educational one: women from more deprived census blocks are more likely to participate in screening than those from the least deprived areas (OR 1.13; 95%CI 1.11–1.16) whereas the educational level showed an indirect gradient with low-educated women being the more adherent (OR 1.40; 95%CI 1.36–1.43). Furthermore, younger women adhered the most to screening whereas those aged 55–59 the least. Women from Highly Developed Country (HDC) and HMCC were 40 and 50% less likely than Italians to participate in the screening, respectively and a regular invitation to screening massively increased the chance of participating (OR 4.93; 95%CI 4.80–5.06).

**Table 1:** Participation rate: descriptive statistics and estimates from univariable logistic models

	Total (N)	Adherent (%)	Odds ratio	95% Confidence intervals	*p* value
**Age group**					
**50–54**	143,227	61,9%	1.00		
**55–59**	131,778	59.2%	0.89	0.88–0.91	<0.001
**60–64**	127,374	59.6%	0.91	0.89–0.92	<0.001
**65–69**	125,608	59.3%	0.90	0.88–0.91	<0.001
**Missing data**	0	0.0%			
**Citizenship**					
**Italian**	480,147	61.8%	1.00		
**HDC**	1733	44.4%	0.49	0.45–0.54	<0.001
**HMPC**	37,418	43.0%	0.47	0.46–0.48	<0.001
Missing data	8689	40.8%			
**Regularity**					
**First exam**	41,347	38,5%	1.00		
**Irregular**	65,222	46.5%	1.39	1.35–1.42	<0.001
**Regular**	312,723	66.8%	3.21	3.14–3.27	<0.001
**Missing data**	108,695	57.0%			
**Educational level**					
**University degree**	49,565	55.0%	1.00		
**High school**	113,771	58.2%	1.14	1.12–1.16	<0.001
**Middle school**	227,447	61.4%	1.30	1.28–1.33	<0.001
**Elementary school/no education**	128,083	62.6%	1.37	1.34–1.40	<0.001
**Missing data**	9121	40.5%			
**Deprivation level**					
**Lowest deprivation**	64,959	57.0%	1.00		
**Low deprivation**	71,851	59.7%	1.12	1.10–1.15	<0.001
**Medium deprivation**	100,425	59.9%	1.11	1.09–1.14	<0.001
**High deprivation**	116,848	61.8%	1.21	1.18–1.23	<0.001
**Highest deprivation**	172,927	60.3%	1.13	1.11–1.16	<0.001
**Missing data**	977	32.3%			
**Total**	527,987	60.1%

**Table 2:** Estimates from multilevel random slope models mutually adjusted for listed variables: (A) participation rate, (B) recall rate, (C) cancer detection rate

	(A) Participation rate			(B) Recall rate			(C) Cancer detection rate		
	Odds ratio	95% Confidence intervals	*p* value	Odds ratio	95% Confidence intervals	*p* value	Odds ratio	95% Confidence intervals	*p* value
	Fixed part								
**Age group**									
**50–54**	1.00			1.00			1.00		
**55–59**	0.74	0.72–0.75	<0.001	0.93	0.89–0.97	<0.001	1.20	0.06–1.35	0.005
**60–64**	0.81	0.79–0.82	<0.001	0.90	0.86–0.94	<0.001	1.52	1.34–1.72	<0.001
**65–79**	0.87	0.86–0.89	<0.001	0.91	0.87–0.95	<0.001	1.93	1.71–2.18	<0.001
**Citizenship**									
**Italian**	1.00			1.00			1.00		
**HDC**	0.59	0.53–0.66	<0.001	0.88	0.65–1.20	0.426	0.50	0.16–1.58	0.24
**HMPC**	0.50	0.49–0.51	<0.001	0.89	0.83–0.95	0.001	0.76	0.62–0.93	0.008
**Exam timing**									
**Further exam**				1.00			1.00		
**First exam**				2.14	2.05–2.23	<0.001	2.26	2.02–2.53	<0.001
**Regularity**									
**First exam**	1.00								
**Irregular**	1.15	1.12–1.19	<0.001						
**Regular**	4.93	4.80–5.06	<0.001						
**Educational level**									
**University degree**	1.00			1.00			1.00		
**High school**	1.13	1.10–1.15	<0.001	0.94	0.89–0.99	0.011	0.89	0.78–1.02	0.088
**Middle school**	1.35	1.32–1.37	<0.001	0.88	0.84–0.93	<0.001	0.84	0.74–0.95	0.006
**Elementary school/no education**	1.40	1.36–1.43	<0.001	0.81	0.76–0.86	<0.001	0.80	0.69–0.93	0.003
**Deprivation level**							1.00		
**Lowest deprivation**	1.00			1.00					
**Low deprivation**	1.06	1.02–1.10	0.004	0.98	0.92–1.04	0.452	1.05	0.89–1.25	0.529
**Medium deprivation**	1.12	1.08–1.16	<0.001	0.96	0.91–1.02	0.175	1.01	0.86–1.18	0.907
**High deprivation**	1.15	1.11–1.18	<0.001	0.94	0.89–1.00	0.038	1.12	0.97–1.30	0.118
**Highest deprivation**	1.13	1.10–1.16	<0.001	0.93	0.88–0.98	0.009	1.08	0.93–1.24	0.316

### Recall rate

Table 3 shows the descriptive statistics and the estimates of the univariable logistic models. Overall, the recall rate was about 5%. The odds of being recalled was the lowest among women aged 65–79 years (OR 0.74, 95%CI 0.71–0.77), those with low education (OR 0.68, 95%CI 0.64–0.72), and those living in the most deprived areas (OR 0.91, 95%CI 0.86–0.96). It was the highest among women from HMPC (OR 1.11, 95%CI 1.03–1.18) and those at their first exam (OR 2.18, 95%CI 2.09–2.26). The Wald test for the null hypothesis that the random coefficient of deprivation is different from zero yields a *p* value of 0.222, suggesting that there is no significant variability in the recall rate among census blocks with a similar deprivation level. The estimates of the random slope multilevel model suggest that individual characteristics matter more than the contextual level of the recall rate (Table 2). After controlling for all variables, the deprivation level did not show a significant effect; instead, education presented a direct gradient with low-educated women being recalled the less (OR 0.81; 95%CI 0.76–0.86). The probability of being called for a further exam after a screening round decreases as age increases. Women from HMPC were about 10% less likely than Italians to be recalled, whereas women at their first exam had 2.14 odds of being recalled (95%CI 2.05–2.23) compared to those at subsequent rounds.

**Table 3:** Recall rate: descriptive statistics and estimates from univariable logistic models

	Total (N)	Recalled (%)	Odds ratio	95% Confidence intervals	*p* value
**Age group**					
**50–54**	88,648	6.3%	1		
**55–59**	77,952	5.3%	0.83	0.80–0.87	<0.001
**60–64**	75,922	4.9%	0.76	0.73–0.80	<0.001
**65–79**	74,529	4.8%	0.74	0.71–0.77	<0.001
Missing data	0	0.0%			
**Citizenship**					
**Italian**	296,635	5.3%	1		
**HDC**	769	5.6%	1.05	0.76–1.44	0.734
**HMPC**	16,099	5.9%	1.11	1.03–1.18	<0.001
**Missing data**	3548	7.8%			
**Exam timing**					
**Further exam**	281,878	4.8%	1		
**First exam**	35,159	9.9%	2.18	2.09–2.26	<0.001
**Missing data**	14	0.0%			
**Educational level**					
**University degree**	27,252	6.6%	1		
**High school**	66,192	5.7%	0.85	0.79–0.90	<0.001
**Middle school**	139,687	5.4%	0.81	0.76–0.85	<0.001
**Elementary school/no education**	80,227	4.6%	0.68	0.64–0.72	<0.001
**Missing data**	3693	7.7%			
**Deprivation level**					
**Lowest deprivation**	37,161	5.7%	1		
**Low deprivation**	43,106	5.5%	0.96	0.90–1.02	0.215
**Medium deprivation**	60,106	5.5%	0.96	0.91–1.02	0.19
**High deprivation**	72,157	5.2%	0.92	0.86–0.96	0.001
**Highest deprivation**	104,205	5.2%	0.91	0.86–0.96	<0.001
**Missing data**	316	6.3%			
**Total**	317,051	5.4%			

### Cancer detection rate

Table 4 shows the descriptive statistics and the estimates of the univariable logistic models. Overall, the detection rate was 0.7%. The odds of being diagnosed with breast cancer was the highest among women aged 65–79 years (OR 1.57, 95%CI 1.41–1.76) and those at their first exam (OR 1.81, 95%CI 1.63–2.01). Citizenship, educational level, and deprivation of the living area were not associated with the recall rate. The Wald test for the null hypothesis that the random coefficient of deprivation is different from zero yields a *p* value of 0.3, suggesting that the deprivation does not present a random effect. As for the recall rate, the estimates of the random slope multilevel model indicate that individual characteristics matter more than the contextual level of the cancer detection rate (Table 2). After controlling for all variables, the deprivation level did not exert a significant effect; instead education showed a direct gradient with low-educated women showing a 20% risk reduction (OR 0.80; 95%CI 0.69–0.93) compared to those more educated. The probability of breast cancer increased with age, and women 65–69 years old had about two times the probability of having a cancer diagnosis (OR 1.93; 95%CI 1.71–2.18) than the younger counterpart. Women from HMPC were about 24% less likely than Italian to be diagnosed with breast cancer whereas those at their first screening exam had 2.26 the odds of getting the diagnosis (95%CI 2.02–2.53) compared to those at their subsequent screening rounds.

**Table 4:** Cancer detection rate: descriptive statistics and estimates from univariable logistic models

	Total (N)	Detected (%)	Odds ratio	95% Confidence intervals	*p* value
**Age group**					
**50–54**	88,645	0.63%	1.00		
**55–59**	77,947	0.67%	1.07	0.95–1.21	0.252
**60–64**	75,918	0.81%	1.29	1.15–1.44	<0.001
**65–79**	74,527	0.98%	1.57	1.41–1.76	<0.001
Missing data	0	0.00%			
**Citizenship**					
**Italian**	296,627	0.77%	1.00		
**HDC**	769	0.39%	0.50	0.16–1.56	0.234
**HMPC**	16,098	0.63%	0.82	0.67–1.00	0.047
**Missing data**	3543	0.76%			
**Exam timing**					
**Further exam**	281,878	0.70%	1.00		
**First exam**	35,159	1.27%	1.81	1.63–2.01	<0.001
**Missing data**	0	0.00%			
**Educational level**					
**University degree**	27,251	0.91%	1.00		
**High school**	66,189	0.78%	0.86	0.74–0.98	0.046
**Middle school**	139,685	0.73%	0.80	0.70–0.92	0.002
**Elementary school/no education**	80,224	0.76%	0.83	0.71–0.96	0.011
**Missing data**	3688	0.77%			
**Deprivation level**					
**Lowest deprivation**	37.159	0.74%	1.00		
**Low deprivation**	43.106	0.80%	1.08	0.92–1.26	0.353
**Medium deprivation**	60.101	0.76%	1.03	0.88–1.19	0.735
**High deprivation**	.154	0.79%	1.06	0.92–1.22	0.441
**Highest deprivation**	104.201	0.75%	1.01	0.88–1.15	0.941
**Missing data**	316	1.27%			
**Total**	317.037	0.77%			

## Discussion

This study examined the impact of contextual deprivation and individual socioeconomic position on screening indicators for women aged 50–69 in Turin, Italy. Both contextual and individual socioeconomic factors were found to affect the participation rate, with individual socioeconomic position having a greater influence. Screening uptake increased with higher levels of deprivation in the residential area and lower educational levels among women. However, the residential context of the women did not significantly impact the recall rate and cancer detection rate, which were primarily influenced by individual educational level. Low-educated women had a 20% lower probability of being recalled or diagnosed with breast cancer. Immigrant women were less likely than Italians to participate in screening and be recalled or diagnosed with breast cancer.

A positive association was found between census block deprivation, educational level, and breast cancer screening participation in Turin. This contrasts with previous studies in Europe and elsewhere that found inequalities in screening uptake based on small-area deprivation^5,9–12^ and educational level^13,14^. One possible explanation of this somehow unexpected gradient is that women living in less deprived census blocks and with high education are more likely to be screened opportunistically and adhere less to organized screening programs. This hypothesis is supported by the results of the PASSI (*Progressi delle Aziende Sanitarie per la Salute in Italia*) survey, a national population-based surveillance system that collects information on population health and modifiable risk factors associated with the development of chronic diseases. According to 2020–21 data for Piedmont, the uptake of organized breast cancer screening is higher among less educated women (49% in those with no education/elementary school; 58% in those with middle school education) than among those with a university degree (45%). Conversely, participation in opportunistic screening follows the opposite educational gradient based, with women with a university degree participating the most (31%) and those with low education participating the least (3.6%). Although these data are not publicly accessible, we have requested access from the Piedmont regional coordinator, and upon obtaining authorization, they have been provided in Supplementary Table 1.

The finding that living in a less deprived census block had a significant effect on the participation rate, though smaller in size than the one of individual Socioeconomic Position (SEP), suggests that the association between community context and screening uptake is at least partly contextual, and not simply compositional^15,16^. In other words, besides the fact that living in a higher SEP census block is associated with a lower screening uptake simply because there are more highly educated women living in those areas (compositional effect), there is something about living in higher SEP small areas that is associated with smaller odds of organized screening adherence (contextual effect). Indeed, as suggested by a wealthy body of research^15^, features of material infrastructures and collective social functioning of local environments may influence health by shaping opportunities and resources available to individuals to promote one’s health capital. Greater availability of private services or cultural and social norms shared among affluent women related to the healthcare system and the use of private services for gynecological issues may impact on the health seeking behaviors. However, with our data, we could not test these pathways and mechanisms, which may need a qualitative approach.

The deprivation level of the census block the woman lives in was not associated with either the recall or the cancer detection rate whereas the individual level of education had a significant direct effect on both screening indicators. As reported in the literature, higher education level seems to be associated with an increased risk of developing breast cancer through many mediating factors such as alcohol use, age at menopause, hormone therapy, parity, and breastfeeding^17,18^. Assuming that the need for further assessment after a screening round is related to the risk of developing breast cancer, the same explanation may apply to our finding of a higher recall rate among highly educated women.

After accounting for both contextual and individual socioeconomic factors, immigrant women were less likely than Italians to participate in the screening as well as to be recalled or diagnosed with breast cancer. These results raise concerns about the equity of the screening process. Reduced access to preventive services among immigrants has been previously reported in Italy and elsewhere^19,20^ and mainly ascribed to potential cultural, communication, healthcare-system-related, and knowledge-related barriers^21^, including a differential awareness of National Health Service cancer screening programs. We could not investigate whether the lower participation rate among immigrant women had an impact on the chance of receiving a diagnosis, but this remains an urgent question to be answered and, potentially, to act upon.

Despite the large sample size and robust statistical methods, there are a few limitations to consider when interpreting the results. First, some explanatory variables had missing data, which could introduce bias. Second, to define the boundaries of the small areas and to obtain a contextual measure of deprivation, we used census data, and therefore, we were bound to census blocks. However, these geographic units cannot be labeled as communities or neighborhoods^22^. Moreover, we cannot exclude a certain degree of exposure misclassification inherent to the deprivation measure we used. Indeed, the deprivation index is based on outdated sociodemographic data, which may not accurately reflect the current social and material deprivation and dilute the association between contextual SEP and the outcomes. There may also be issues with multicollinearity between small-area deprivation and individual educational level because the index contains information on education leading to a high correlation between them. This may make it difficult to distinguish their independent effects on the outcomes and to appreciate the contextual effect, if any, once the individual level is introduced in the model. Screening behavior information was incomplete for women screened before 2010, but assuming that the regularity does not change much over time, the risk of bias should be minimal. The study was conducted in an urban setting, so the results may not be generalizable to other contexts. Lastly, the administrative databases used for this study do not contain information on religious and cultural background, preventing us from studying the impact of such variables on adherence to breast cancer screening.

Our findings show that participation in organized screening increases among individuals with lower educational levels and higher levels of deprivation. Highly educated women have a higher likelihood of being recalled or diagnosed with breast cancer, while immigrant women have a lower likelihood of participating in screening and being recalled or diagnosed. These findings highlight the need for policies that encourage the participation of highly educated women in organized breast cancer screening programs, which align with established guidelines and weight risks and benefits in terms of radiation and over-diagnosis. Furthermore, the lower screening uptake among immigrant women raises concerns about the fairness of screening access and emphasizes the importance for the healthcare sector and researchers to identify and address existing barriers in order to promote equitable policies.

## Methods

### Study population

*Prevenzione Serena* is a cancer screening program in the Piedmont Region that aims to prevent and detect breast, cervical, and colorectal cancer. The breast cancer screening program invites all women living in Piedmont, including both Italian and foreign residents, aged between 50 and 69 years, who are registered with the regional health registry.

Italy operates a universal health system, based on the Beveridge model, which provides healthcare services to all citizens and individuals with regular residence permits. However, to access these services, registration with the health registry of the territory of residence is required. This register is used to identify the population eligible for organized screening invitations.

The study focuses on women living in Turin between 2010 and 2019. A total of 527,987 invitations were sent to eligible women during this period. The same woman could receive multiple invitations as mammograms are repeated every two years. In the Supplementary Fig. 1 a flow chart of the data selection process is provided.

### Data sources

Data come from the Turin Breast Screening Program (TBSP) database and the Turin Longitudinal Study (TLS) database. The two sources were linked at an individual level via a unique anonymous key. The TBSP contains, along with a unique patient’s ID, variables on the participation type (whether the woman is adherent or not, spontaneous or she had a recent mammography), the year the screening was done, the sequence of the screening round for each invite. This dataset was used to derive the screening outcomes. The TLS is a system of integrated data on health outcomes, demographic, and socioeconomic information that draws upon the municipal civil register, the census data, and the health information system for the residents of the city of Turin^23^. It provided information on the contextual and individual socioeconomic position of the study population.

### Breast cancer screening indicators

The participation rate, the recall rate, and the cancer detection rate are indicators that span throughout the entire screening process and reflect the logistic organization, the process, and the performance of the screening program^24^ (Table 5).

**Table 5:** Definition, aim, and calculation of screening indicators

Indicator	Definition	Aim	Calculation
**Participation rate**	Number of women who have a screening test over all women invited to attend the screening (invited women are those eligible at the date of the invite)	To assess the impact and efficiency of the program in reducing mortality through a direct and proportional effect on the outcome	$\frac{{\rm{number}}\,{\rm{of}}\,{\rm{women}}\,{\rm{who}}\,{\rm{have}}\,{\rm{a}}\,{\rm{screening}}\,{\rm{test}}}{{\rm{number}}\,{\rm{of}}\,{\rm{women}}\,{\rm{invited}}\,{\rm{to}}\,{\rm{screening}}-{\rm{unreturned}}\,{\rm{invitations}}}$
**Recall rate**	Number of women recalled for further assessment as a proportion of all women who had a screening examination	To determine, along with other indicators, the specificity of the first level of the program. A further exam is needed to clarify what has been classified as an anomaly at the first level	$\frac{{\rm{number}}\,{\rm{of}}\,{\rm{women}}\,{\rm{recalled}}\,{\rm{for}}\,{\rm{further}}\,{\rm{assessment}}}{{\rm{number}}\,{\rm{of}}\,{\rm{women}}\,{\rm{who}}\,{\rm{had}}\,{\rm{a}}\,{\rm{screeningexamination}}}$
**Detection rate**	Number of pathologically-proven malignant lesions of the breast detected in a screening round per 1000 women screened in that round	Process indicator that aims at indirectly estimating the efficiency of the screening program	$\frac{{\rm{number}}\,{\rm{of}}\,{\rm{malignat}}\,{\rm{breast}}\,{\rm{lesions}}\,{\rm{detected}}\,{\rm{in}}\,{\rm{a}}\,{\rm{screening}}\,{\rm{round}}}{{\rm{number}}\,{\rm{of}}\,{\rm{women}}\,{\rm{screened}}\,{\rm{in}}\,{\rm{that}}\,{\rm{round}}}$

### Exposures and other variables

The SEP was measured at both contextual and individual levels. The deprivation index, based on the 2011 census data for the city of Turin, is an area-level indicator of SEP. It measures social and material deprivation using five standardized variables: low education, unemployment, non-home ownership, single-parent family, and house overcrowding^25^. The index was divided into five quintiles, ranging from 1 (less deprived) to 5 (more deprived). The women’s educational attainment is the individual-level indicator of SEP. The educational level is stable over time and, being empirically correlated with other variables such as income, status and class, it is good proxy of the socioeconomic position^26^. It was defined as the highest attained qualification and classified into four levels: no education or elementary school, middle school (or junior high school), high school, and university degree. Other variables of interest were age, citizenship, and past screening behaviors. Age was classified into 5-year age bands: 50–54, 55–59, 60–64, and 65–69. Citizenship was defined as Italian, from HDC, and from HMPC. Regularity of invitation is a variable that indicates whether a woman is regularly invited to the screening program or not, the latter happening if, from 2010 to 2019, she was invited to screening after 1000 days from her last call more than 65% of the time. The First Exam is a variable that indicates whether a woman is at her first or subsequent screening round.

### Statistical methods

The main objective of this study is to assess the association between indicators of screening performance and process and the women’s contextual and individual SEP. To test this hypothesis a multi-step analytical approach was followed. For each outcome, we first estimated logistic univariable models to describe the association between the screening indicators and the individual (educational level, age, citizenship, regularity of invitation or first exam) and contextual (deprivation index at census block level) characteristics using STATA V.15. Secondly, we run multilevel models. The reason behind the use of multilevel models to test our hypothesis is that women living in the same small area, i.e. the census block, might show a higher intraclass correlation among covariates as well as outcomes than those living in another census block. Indeed, the multilevel approach allows us to account for the nested structure of the data (women are nested in small areas) and to correctly estimate the standard error therefore providing non-biased estimates. We run a random slope model adjusted for the women’s individual characteristics and for the deprivation index at the census block level. To have a less biased estimation, a first estimation using first-order marginal quasi-likelihood (MQL1) was used to obtain a starting value for a second-order quasi-likelihood method (PQL2). The second estimation using PQL2 is needed since MQL is known to produce estimates which are biased downwards in some cases. The final random slope model will not present a random intercept because, having a census section one and only one deprivation value, adding a random intercept results in a non-convergence due to collinearity^27^. Multilevel models were estimated with MLwiN 2.36 using RIGLS. An approximated Wald test was used to test the random effect, an approximated Wald test, and a chi-square test on the residual to test the variance component of the random effects, as suggested by Hox^28^.

### Supplementary information

Supplementary information

## Data Availability

Raw data cannot be made freely available because of restrictions imposed by the Ethical Committees which do not allow open/public sharing of data on individuals. However aggregated data are available for other researchers, on request. Requests should be sent to the corresponding author.

## Code availability

In an effort to support transparency and reproducibility in research, we could make available on request the code utilized in the methodologies presented within this paper.
