# Supplementary material for: Contextual and individual inequalities in breast cancer screening participation and outcomes in Turin (North-West Italy)
Source: NPJ Breast Cancer. 2024 Jun 27;10:51. doi: 10.1038/s41523-024-00660-4 (PMC11211318; doi:10.1038/s41523-024-00660-4)

Supplementary table 1 - Participation to breast cancer screening in Piedmont by type of screening

**Participation to organized breast cancer screening by socioeconomic characteristics - Piedmont 2020-2021**		**%**	**95% CI**	
**Age**	50-59	51.5%	46.2%	57.4%
	60-69	54.5%	47.6%	61.3%
**Cohabitation**	no	56.3%	47.7%	64.4%
	yes	51.5%	46.3%	56.6%
**Married**	no	58.0%	49.0%	66.4%
	yes	51.0%	46.0%	56.1%
**Education**	no education/elementary school	48.8%	29.5%	68.4%
	middle school	57.9%	49.7%	65.6%
	high school	52.1%	45.7%	58.4%
	university degree	45.4%	33.5%	57.9%
**Economic difficulties**	many	42.8%	25.8%	61.6%
	some	52.2%	43.2%	61.1%
	no difficulties	54.7%	49.5%	59.8%
**Nationality**	Italian	53.6%	49.2%	58.0%
	foreigner	49.0%	30.7%	67.5%

**Participation to opportunistic breast cancer screening by socioeconomic characteristics - Piedmont 2020-2021**		**%**	**95% CI**	
**Age**	50-59	20.6%	16.2%	25.8%
	60-69	10.7%	7.1%	15.8%
**Cohabitation**	no	9.8%	5.9%	16.1%
	yes	19.2%	15.4%	23.6%
**Married**	no	8.5%	4.9%	14.2%
	yes	19.2%	15.5%	23.6%
**Education**	no education/elementary school	3.6%	0.5%	21.7%
	middle school	6.9%	4.1%	11.3%
	high school	19.5%	14.9%	25.1%
	university degree	30.6%	20.6%	42.9%
**Economic difficulties**	many	20.5%	9.3%	39.4%
	some	5.5%	2.7%	11.1%
	no difficulties	20.1%	16.2%	24.8%
**Nationality**	Italian	17.5%	14.2%	21.3%
	foreigner	1.9%	0.3%	12.6%

Data source: Italian Behavioral Risk Factor Surveillance System PASSI - https://www.epicentro.iss.it/passi/

Total participation to breast cancer screening (either organized or opportunistic) by socioeconomic characteristics - Piedmont 2020-2021		%	95% CI	
**Age**	50-59	72.6%	67.0%	77.5%
	60-69	65.7%	58.8%	71.9%
**Cohabitation**	no	66.8%	58.6%	74.1%
	yes	70.7%	65.4%	75.5%
**Married**	no	67.2%	58.5%	74.8%
	yes	70.4%	65.4%	74.9%
**Education**	no education/elementary school	52.3%	32.2%	71.8%
	middle school	64.8%	57.0%	71.8%
	high school	71.7%	65.3%	77.3%
	university degree	77.1%	65.7%	85.5%
**Economic difficulties**	many	63.3%	44.4%	78.8%
	some	58.0%	49.1%	66.5%
	no difficulties	75.1%	70.4%	79.3%
**Nationality**	Italian	71.4%	67.0%	75.4%
	foreigner	50.9%	32.4%	69.1%

Data source: Italian Behavioral Risk Factor Surveillance System PASSI - https://www.epicentro.iss.it/passi/

Supplementary Figure 1 - Flow chart of the data selection procedure

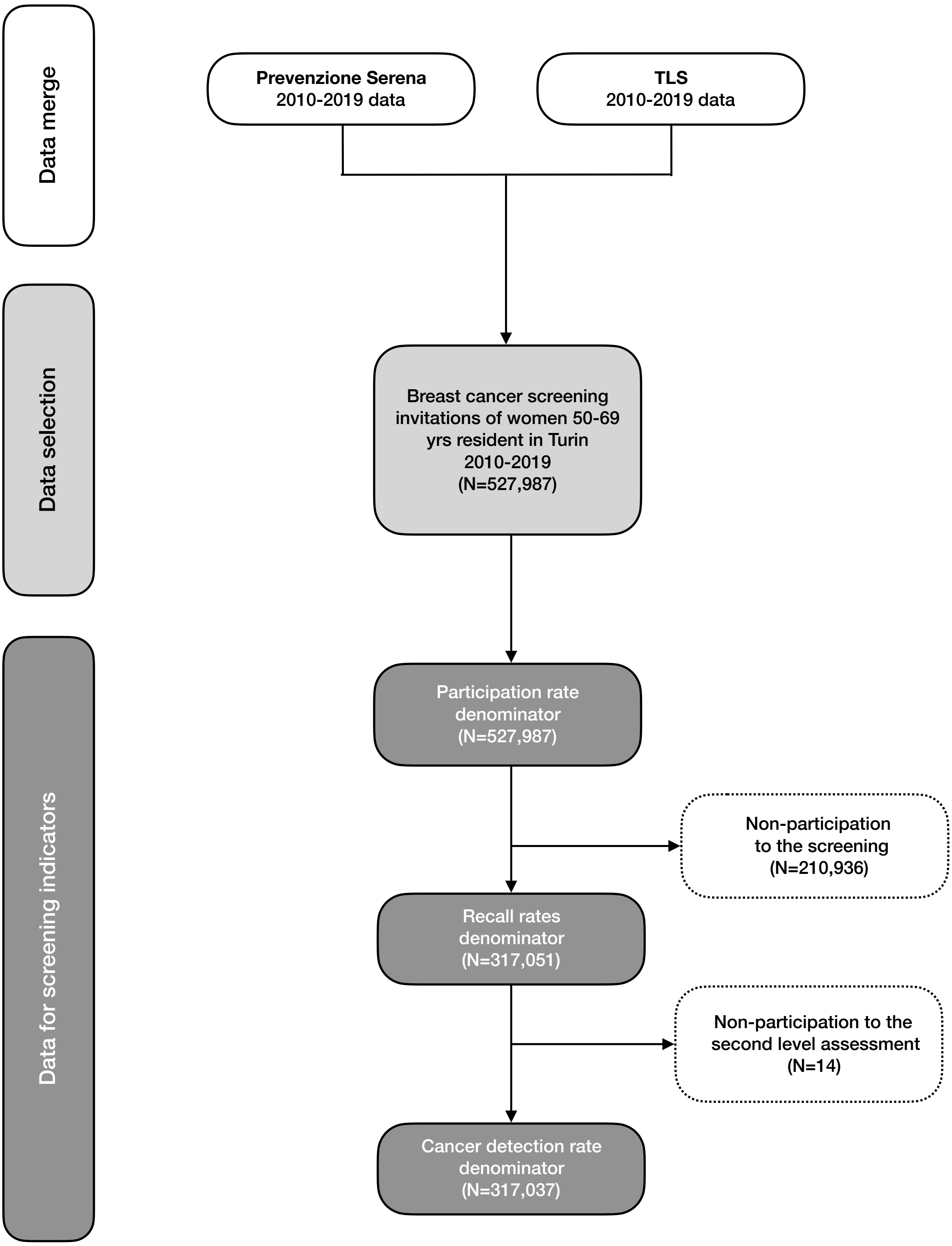

Supplement: Supplementary file 1 — Supplementary information [file 41523_2024_660_MOESM1_ESM.pdf]
